# Photocatalytic degradation of methylene blue dye by ZnO nanoparticle thin films, using Sol–gel technique and UV laser irradiation

**DOI:** 10.1038/s41598-024-76938-1

**Published:** 2024-11-06

**Authors:** Diaa Atta, Hanan A. Wahab, M. A. Ibrahim, I. K. Battisha

**Affiliations:** 1 Spectroscopy Department-Physics Research Institute, National Research Centre (NRC), 33 El-Bohouth St., Dokki, Giza 12622 Egypt; 2 Nonlinear Optical Properties and Fluorescence Unit, National Research Centre (NRC), 33 El-Bohouth St., Dokki, Giza 12622 Egypt; 3 Solid State Physics Department, Physics Research Institute, National Research Centre (NRC), 33 El-Bohouth St., Dokki, Giza 12622 Egypt; 4 Electric and Dielectric Materials Measurement Unit, National Research Centre (NRC), 33 El-Bohouth St., Dokki, Giza 12622 Egypt; 5 Molecular Modeling and Spectroscopy Laboratory, Centre of Excellence for Advanced Science, National Research Centre, 33 El-Bohouth St., Dokki, Giza 12622 Egypt

**Keywords:** Methylene blue industrial waste removal, Zinc oxide nanoparticle, Photo-catalysis, Laser spectroscopy, Laser Irradiation, And B3LYP/6-311g(d,p), Environmental sciences, Environmental chemistry

## Abstract

The focus of the current work is the study of the effect of the photo-catalytic activity of ZnO nanoparticles. The photocatalytic destruction of methylene blue dye, a common water contaminant, was used to assess the photocatalytic efficiency of the ZnO nanoparticles from its aqueous solution by using ZnO nanoparticles thin film under UV light and laser irradiation. Sol–gel methods prepared ZnO nanoparticle thin films. X-ray diffraction and a field-emitted scanning electron microscope were utilized to examine the structure of the produced ZnO nanoparticles. An extended characterization by laser-based fluorescence and UV–visible spectroscopic techniques. The effects of operational parameters such as photo-catalyst load and contact time on photocatalytic degradation of methylene blue were investigated. The recent study’s findings showed that irradiation with a UV laser increases with power density 25 µW/cm^2^, the photo-catalytic rate. The UV spectra show decay for the band at 664nm decreased and the concentration of M.B. in monomer form decayed to 26% of the original concentration in 24 h, while the band at 612 which is related to the dimer M.B. molecules was not affected. The laser irradiation did the same for monomer M.B. molecules in only 3 h, while the dimer decreased to 28% of its original concentration. The reaction mechanism has been discussed by molecular modelling. Quantum mechanical calculations at B3LYP/6-311g(d,p) level indicated that methylene blue changed from dimers to monomers in the existence of ZnO. The current results present a method for degrading M.B. not only in wastewater but also in the industrial waste scale.

## Introduction

A promising technique for treating wastewater contaminated with organic and inorganic contaminants has been identified as photo-catalytic degradation. In recent years, there has been an increase in the number of researchers who have become interested in the method as a technique for removing persistent water contaminants such as dyes and pesticides^1–3^. On the other hand, these techniques also produce solid wastes that need to be further treated as secondary pollutants, such as sludge and hazardous gases^4^.

In recent years, one of the most appealing research areas has been the production of several semiconductor nanomaterials for the removal of artificial textile contaminants from wastewater^5^. Photo-catalysis, using nano-sized semiconductors as photo-catalysts, is a recently emerging, clean, and cost-effective alternative for the large-scale treatment of water polluted with dyes and other organic compounds^6–8^.

Zinc oxide (ZnO) semiconductors have many benefits, particularly high photo-catalytic efficiency, high photosensitivity, non-toxicity, relatively inexpensive, and environmental friendliness^9,10^. The physical, chemical, and optical characteristics of ZnO nanocrystals have drawn a lot of interest since they are useful for heterogeneous catalytic applications, sensors, ultraviolet (UV) photo-detectors, etc.^11–13^.

Methylene blue (M.B.) is a heterocyclic aromatic synthetic form that can be utilized as both a pharmaceutical compound and a dye. It feeds water bodies via effluent from pharmaceutical, paper, and textile industries and hurts aquatic life and the ecosystem. Methylene blue in drinking water is harmful to one’s health because it may irritate the skin and eyes. Moreover, nausea, hemolytic anaemia, abdominal pain, and vomiting may be caused. Therefore, its removal from the polluted water is regarded as one of the most important issues.^14^

M.B. is a member of the thiazine dyes. It is constructed from benzene rings with nitrogen and an electron-rich sulfur atom. M.B., like most synthetic dyes, is one of those molecules that cannot be degraded naturally.^14^

In general, the presence of any dyes in the aquatic media causes colouration in that media. Staining the aquatic media leads to a decrease in the sunlight amount through the water; this is harming the aquatic creatures and affecting the ecology of the deep water^15^. On the other hand, the accumulation of M.B. in living organisms causes dermatological diseases and several mutations, as the M.B. accumulated in the body increases the cancer probability^16,17^.

Even M.B. could be used as a monitoring probe or as a drug for some blood haemoglobin disorders. It has been indicated as an incredibly hazardous drug, with an overdose quickly transforming ferrous iron from reduced haemoglobin to ferric iron, resulting in methemoglobin creation.

M.B. aggregation occurs when its concentration reaches a few µM or higher. The aggregated M.B. molecules transform into inactive molecules because of the sharing of the free electrons, causing saturation, the matter that affects the photo-degradation process to overcome its pollution hazard^18,19^. Recently, surface activation and quenched dye molecules reactivation and laser ablation have been used intensively^19,20^. Reactivation of M.B. dimers could be done by high energy ablation, which could break the M.B. dimer.

Spectroscopic tools such as FTIR^21–23^, Raman^24–26^, single-molecule and time-resolved spectroscopy^27–29^, and molecular modelling play very important roles in vast spectra of scientific work from assigning archaeological and geological objects^30–32^ to medical studies^33,34^.

Molecular modelling could confirm and explain the chemical reaction mechanisms, especially if DFT quantum theories have been applied^35^. Spectrophotometry and emission spectroscopy are the cornerstones in the spectroscopic work. In the field of photo-degradation, spectrophotometry and absorption spectra have been used exclusively^36–39^.

Several studies and research suffer from the presence of the dimer molecules by using many types of catalysis like nano-TiO_2_ films^40^ and the utilization of gold-loaded hydroxyapatite nanoparticles^41^ and much other work all suffer from the presence of dimer and its resistance to the catalysis to be degraded as the monomer molecules especially if the M.B. waste is in industrial scale.

The originality of this work is the utilization of UV-Laser to overcome the formation of M.B. Dimers in the high concentration M.B. which is found in industrial waste and very hard to clean the waste from.

The aim behind this work is to establish a new economic surface with a few nano composite ZnO thin films on such glass substrate able to clean the aquatic media from the presence of dangerous pollutants such as M.B. The second goal is even more important than the first one, which is employing an ultra-clean method (UV laser) to overcome the aggregation of the M.B. that takes place if the M.B. concentration becomes higher than a few micro molars, i.e., using that combination from UV-laser and few layers of ZnO Nano layers. To understand the mechanism of interaction, M.B. model molecules were subjected to molecular modelling at DFT: B3LYP/6-311g(d,p) level.

## Experimental

### ZnO nanoparticles thin film (ZnOTF) preparation

The substrates should be first pre-treated by cleaning the substrate in a sonication has ethanol for 15 min. The ZnO nanoparticles preparation has been explained in detail in previous work^42^. The ZnO solution, zinc acetate dehydrate from Oxford was dispersed in a combination of 2-methoxyethanol (2ME) from Merch and mono-ethanolamine (MEA) from Oxford at room temperature and stirred for two hours at 50 °C. After stirring, the mixture was incubated at room temperature for 24 h; then, the mixture was spun at a speed of 3000 rpm for 30 s to coat the surface of the glass substrates with nano-crystalline ZnO.

Multiple coats of the solution were applied and dried at room temperature in open air, and then the zinc acetate dehydrate was decomposed into ZnO nanoparticles by annealing at 500 °C.

### Characterization

The prepared ZnOTFs were subjected to XRD to identify their crystalline composition.

The XRD patterns have been created by using a Philips X-ray diffractometer operated at 40 kV and 30 mA and equipped with monochromatic Cu Kα radiation of wavelength = 1.54056 Aº.

Scherrer equation^43^ was used to calculate the crystallite size G:

1 $${\it\text{G}} = {\text{K}}{\lambda}/{\text{Dcos }}({\text{q}})$$

where the wavelength is λ, the Scherrer constant is K = 0.9, and the full width (in radians) of the peak intensity at half maximum is D, symbolic as (FWHM). The value of G was confirmed by using the U-fit program.

The produced samples’ coarse and fine microstructures have been recorded using the FESEM model Quanta FEG 250 from FEI, USA.

### Photo-catalysis experiment

The photo-catalysis experiment was done by irradiating the samples to UV waves in a dark room equipped with UV-A, UV-B, and UV-C lamps with a total power of 120 W.

#### UV laser irradiation setup

In the case of investigating the effect of directed energy instead of diffused energy, an Argon ion laser tube from Coherent, USA, has been utilized. The Argon ion tube cavity was equipped with UV-reflecting mirrors instead of the visible pair, and then the back prism was adjusted to reflect only the 305 nm line. The laser power was adjusted to 5 mW employing the attenuating wheel. The laser beam has been expanded using a homemade beam expander to enlarge the laser beam diameter to cover the whole area of the ZnO-coated glass. The used optics in the beam expander have been tested for transmitting the laser line at 305 nm using spectrophotometer model V730 from Jasco-Japan.

The beam diameter at the sample reached 5 cm. The power density, in this case, could be calculated as^44^:

$$\begin{gathered} power\;density = \frac{total\;laser\;power}{{crosssection\;area}} \hfill \\ \quad \quad \quad \quad \quad \quad \;\;\, = \frac{{5 \left( {{\text{mW}}} \right)}}{{6.25 \pi \left( {{\text{cm}}^{2} } \right)}} = 0.25\frac{{{\text{mW}}}}{{{\text{cm}}^{2} }} \hfill \\ \end{gathered}$$

#### Spectrophotometry

The previously mentioned spectrophotometer has been utilized to measure the absorbance of the M.B. Equal piece of glass substrate coated with 3 and 6 layers (with area 6.25 cm^2^) of ZnOTF have been dipped in a 50 ml glass beakers, with its coated side upward, then an equal amount (25ml) of M.B. of concentration 14.8 µM poured in each beaker.

All beakers were incubated in a darkroom without any irradiation for an hour with continuous stirring to avoid adsorption and desorption of M.B. to the ZnOTFs, then 2ml was were stored in an Eppendorf tube as zero time sample before irradiating the substrates with UV waves. 2ml of M.B. solution was withdrawn in the Eppendorf tube after starting the irradiation with 5 min, 10 min, 30 min, 60 min, 120 min, 180 min, and 300 min. Moreover, after 24 h, the last sample was withdrawn. The whole experiment was done inside a dark room equipped with 3 UV lambs in the A, B, and C regions, also continuous stirring during the experiment time. An equal amount of M.B. was exposed to the UV light without dipping anything as the control sample, and only a single sample was withdrawn after 24 h.

### Calculations details

All the studied model molecules were subjected to calculations using G09 softcode^45^, which was implemented in the personal computer at molecular spectroscopy and modelling unknit at the National Research Centre. Both total dipole moment and HOMO/LUMO band gap energy were calculated using the B3LYP/6-311g(d,p)^46–49^ model. Molecular electrostatic potential was mapped at the same level of theory.

## Results

**Fig. 1:**
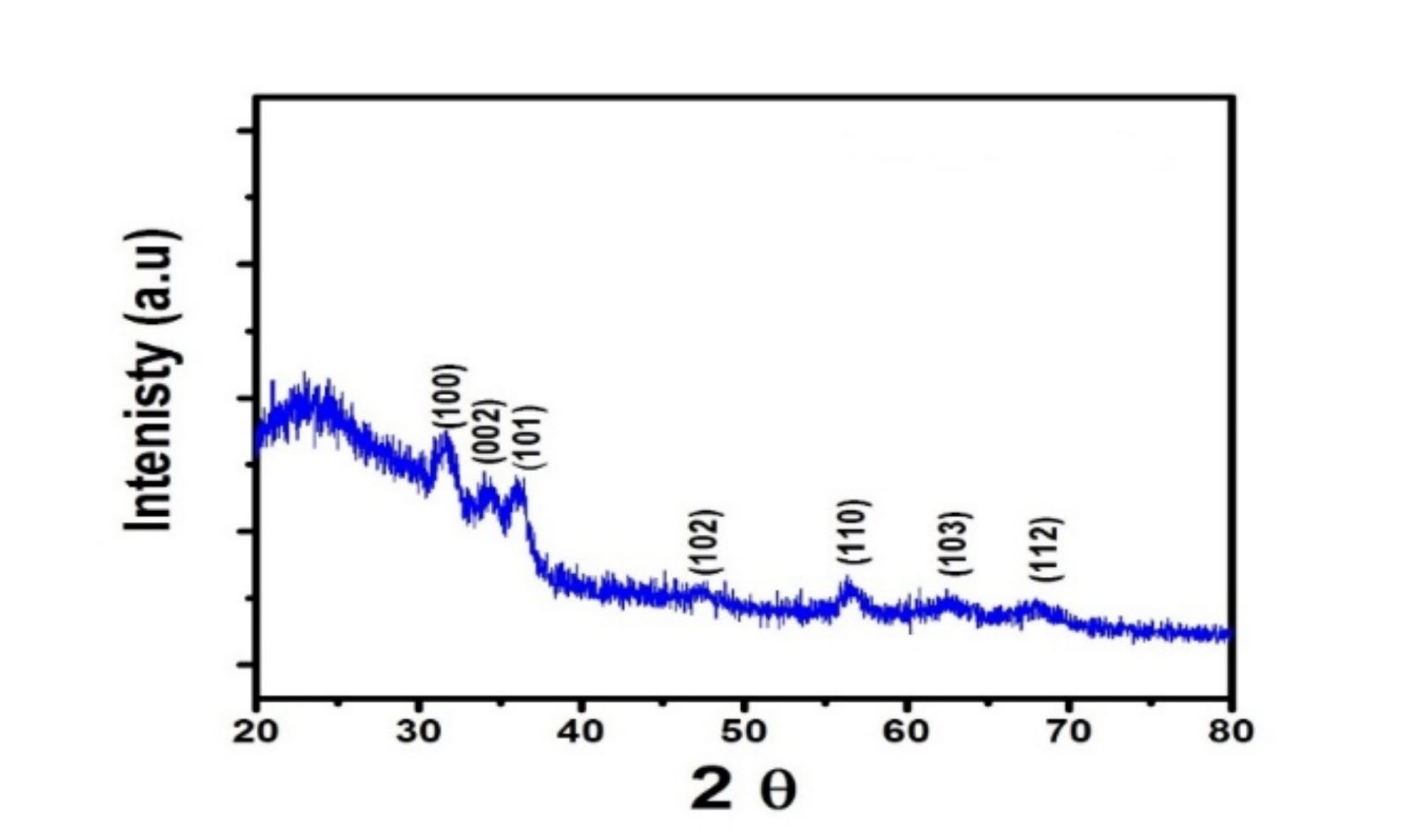
XRD pattern of ZnOTF, annealed at 500 °C.

XRD has been utilized to determine the crystalline architecture of ZnOTF. Figure 1 depicts the XRD pattern of the spin-coated sol–gel ZnO photo-catalyst thin film.

**Fig. 2:**
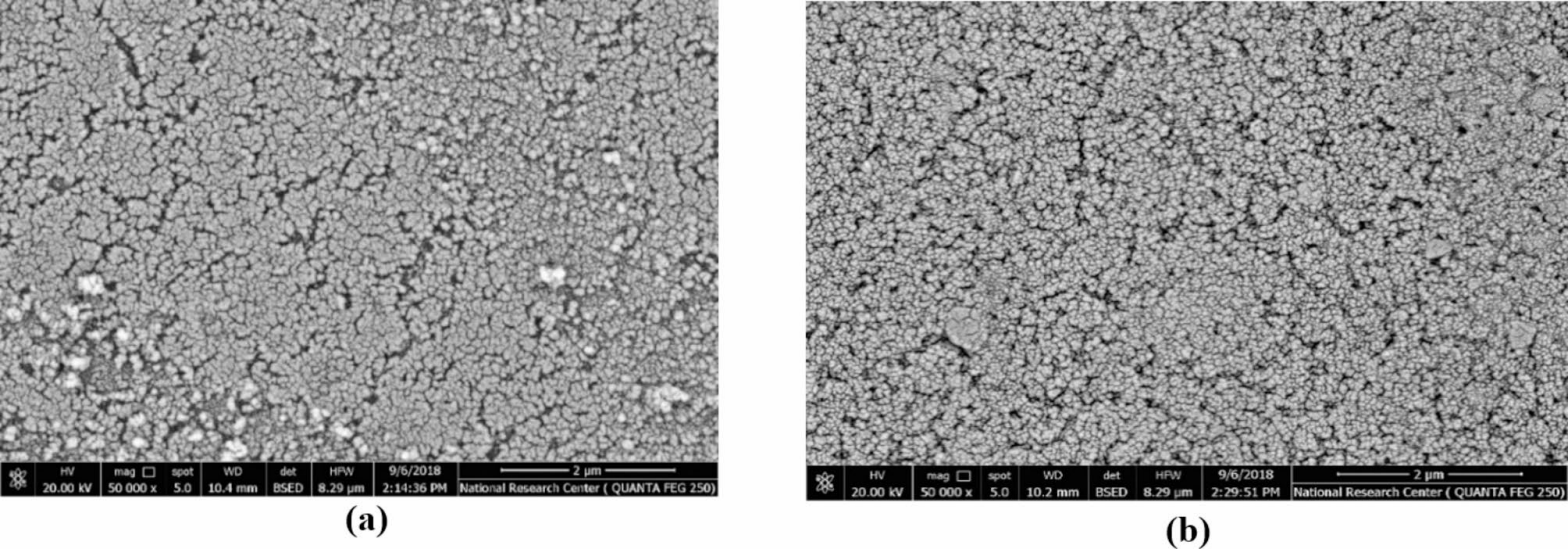
FESEM of ZnOTF (**a**) with three layers, and (**b**) 6 layers, annealed at 500 °C.

Figure 2a, b represents the thin film FESEM of the ZnOTF, both (a) 3 layers and (b) 6 layers, respectively.

**Fig. 3:**
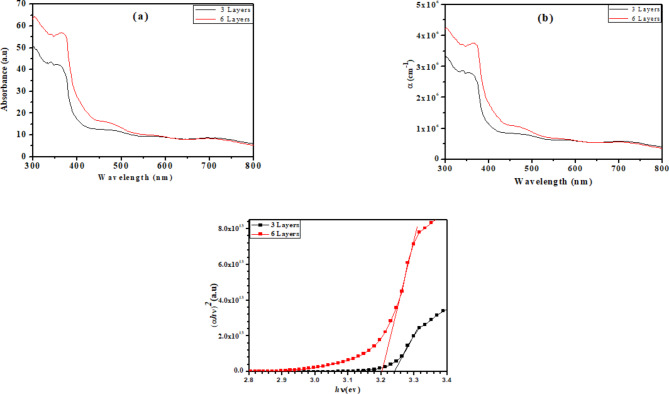
(**a**) The absorbance spectra, (**b**) The absorption coefficient change against the wavelength, and (**c**) the Tauc plot for 3-layers, and 6-layers of ZnOTFs.

The optical band structure has been presented in Fig. 3.

**Fig. 4:**
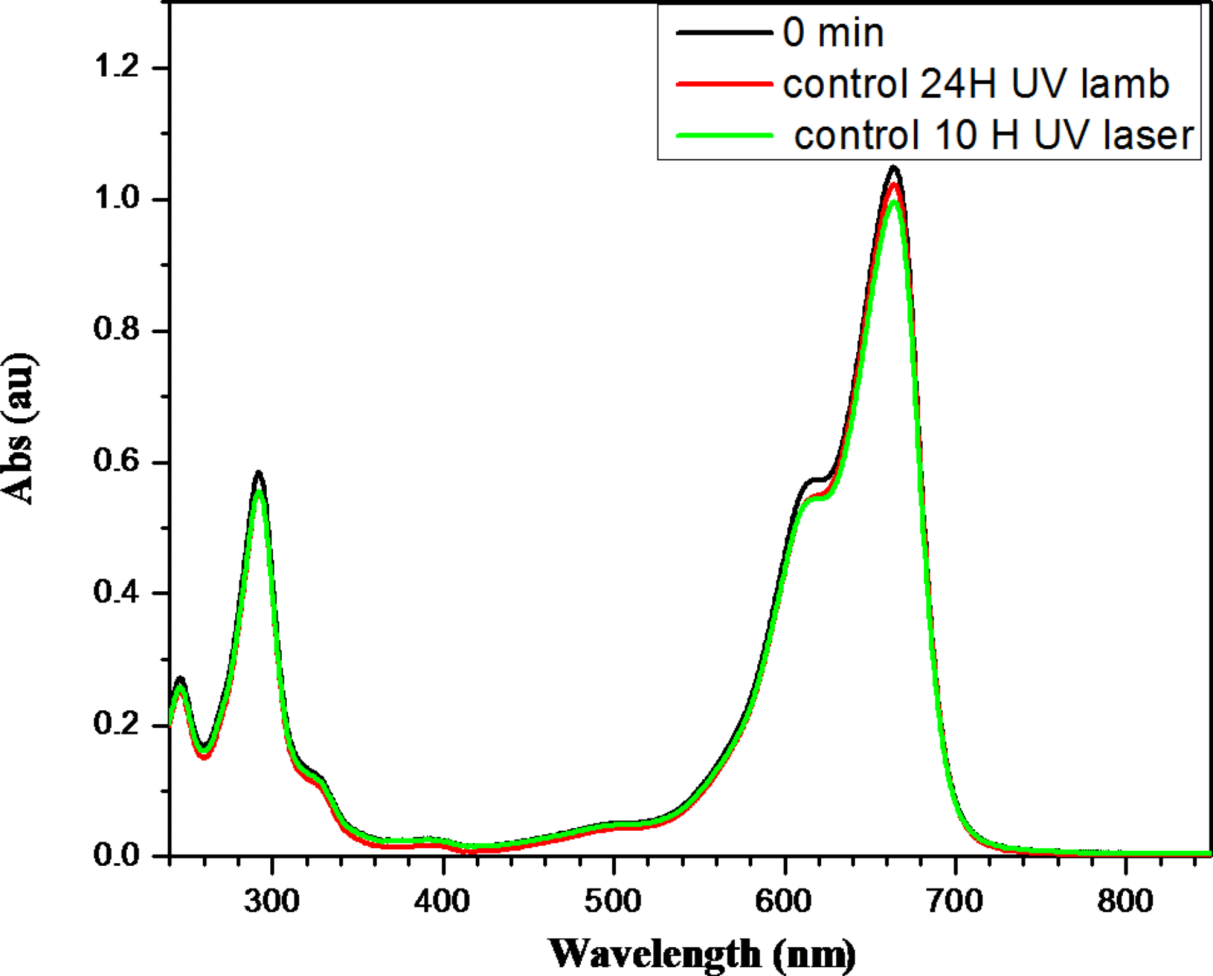
The absorption band of the sample before immersing the ZnOTF (black line), and that was irradiated with UV lamb (red line); also, when illuminated with 305 nm laser expanded to 6.5 cm^2^, it is clear that the UV irradiation nearly had no effect the absorption bands.

Figure 4 presents the effect of the UV radiation without the synthesized ZnOTF.

**Fig. 5:**
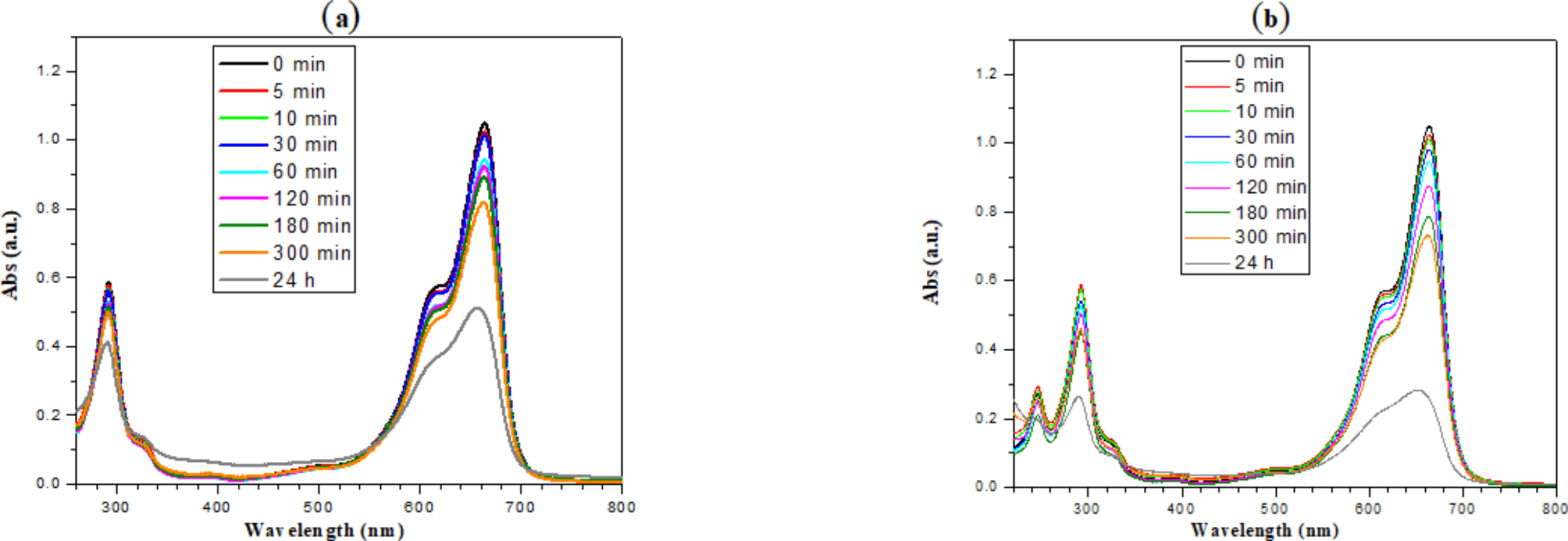
The absorption bands of the samples exposed to (**a**) 3 layers and (**b**) 6 layers of ZnOTF under UV_A, B, and C_ lamb in a dark room, withdrawn after 0, 5, 10, 30, 60, 120, 180, 300 min and 24 h (control sample).

After applying the UV radiation from UV lambs (a, B, and C type) on the ZnOTF slides immersed in the M.B. solutions, the absorption bands have been recorded and overlaid for both 3-layer and 6-layer systems and are presented in Fig. 5a, b.

**Fig. 6:**
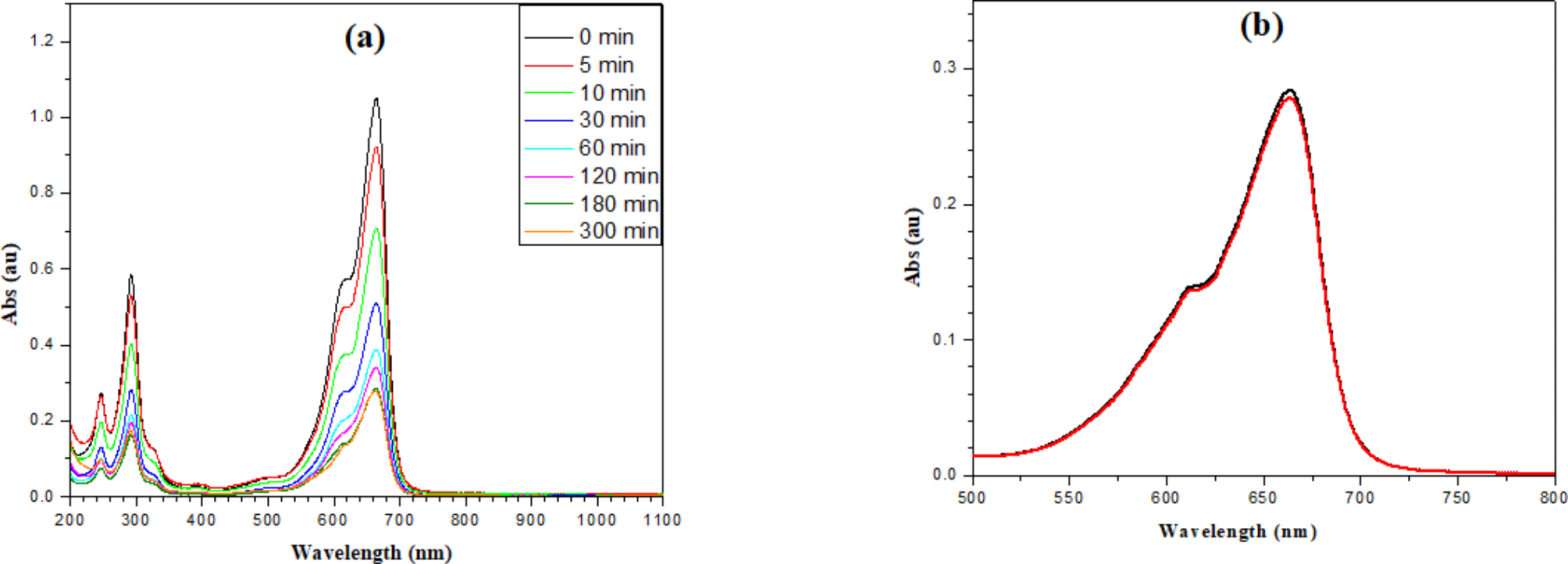
(**a**) The absorption bands of the samples exposed to 6 layers of ZnOTF under 305nm laser irradiation with a power of five mW in the darkroom, withdrawn gradually after 0, 5, 10, 30, 60, 120, 180, and 300 min, (**b**) a comparison between the photodegradation after 180 min in the beginning (red line) of the experiment and after storing for 1 year in 4 °C under irradiation with 305nm laser with power five mW (black line).

PL measurements have been done for the same samples that tested with spectrophotometry after exciting them with a 635 nm laser, as presented in Fig. 6.

It is clear from the previous measurements that the 6-layer ZnOTF has a stronger photodegradation effect on the Prepared M.B. solution. Hence, the UV laser has replaced the UV lamp in an attempt to enhance the photodegradation process.

A comparison between the photodegradation after 180 min after one year and that at the beginning is presented in Fig. 6b.

**Fig. 7:**
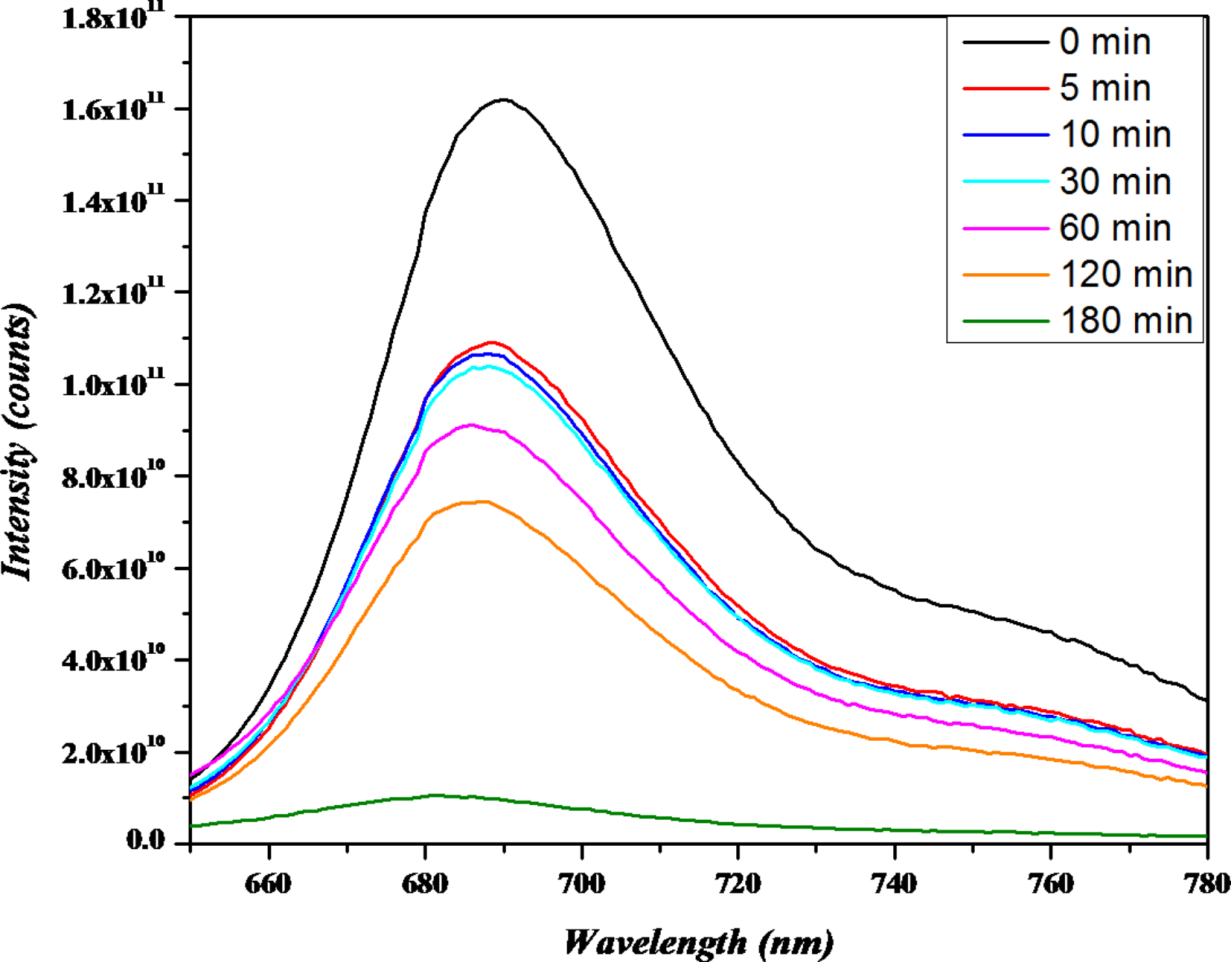
The photoluminescence spectra of the samples exposed to 6 layers of ZnOTF Irradiated with the 305 nm laser with power five mW in the darkroom, withdrawn after 0, 5 10, 30, 60, 120, and 180 min, excited with a picosecond pulsed laser source at wavelength 635nm.

The experiment has been done depending on the 6-layer system as it gives the best results with the normal UV lamb, as presented in Fig. 7.

**Fig. 8:**
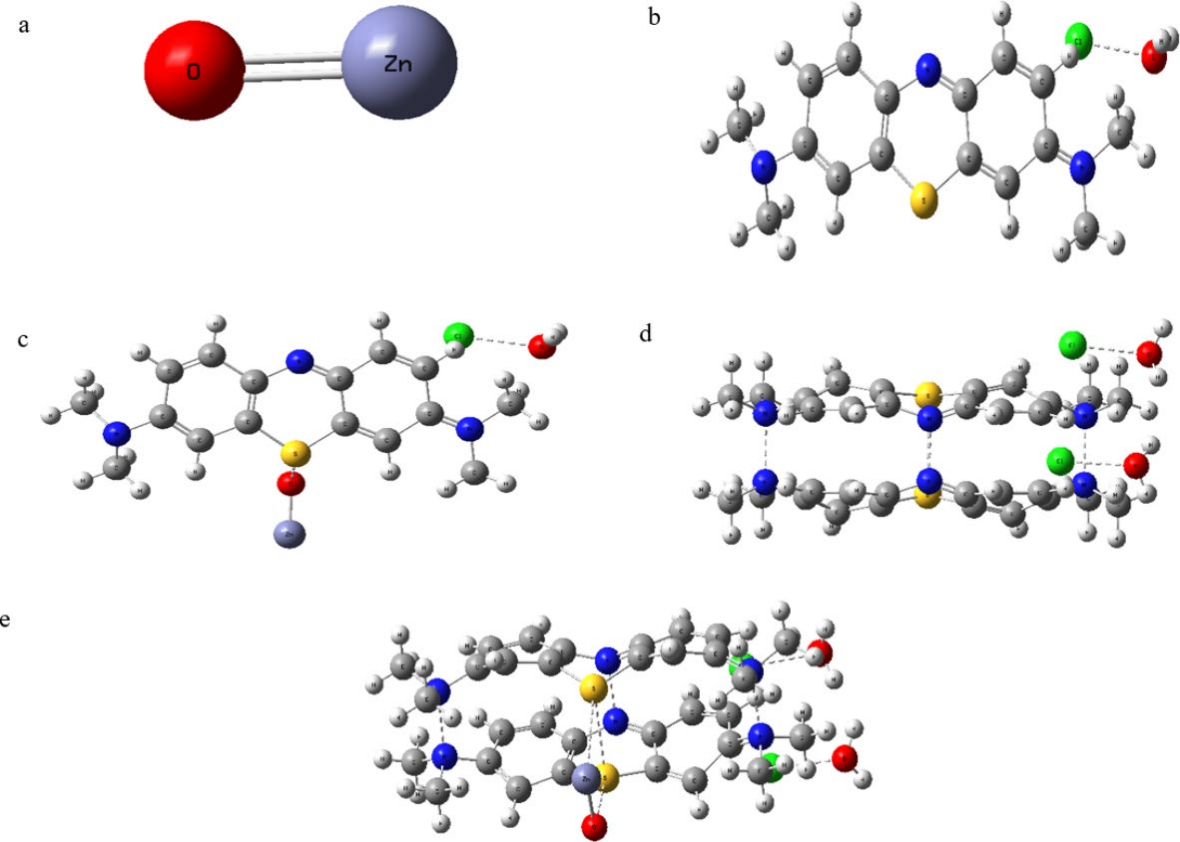
The studied model molecules for (**a**) ZnO, (**b**) Methylene blue hydrated with one water molecule, (**c**) Methylene blue hydrated with one water molecule interacted with ZnO, (**d**) Methylene blue dimer hydrated with two water molecules, and (**e**) Methylene blue dimer hydrated with two water molecules interacted with ZnO.

Molecular modelling could be used to understand the mechanisms of chemical reactions^41^, so to understand the presented photocatalytic reaction mechanism, hydrated M.B. monomer and Dimer in the presence/ absence of the ZnO have calculated as it is presented in Fig. 8.

## Discussion

The identical ZnO thin film’s XRD pattern presented in Fig. 1 shows diffraction peaks at 2θ = 31.8, 34.5, 36.5, 47, 56.6, and 63.0, respectively, corresponding to crystal planes (100), (002), (101), (102), (110), and (103). The fact that no other impurities were found in the diffraction peaks indicates that the compounds that were applied to the slid surface are purely ZnO.

Because all diffraction peaks could be indexed to ZnO with the hexagonal wurtzite structure, the synthesized ZnOTFs have even been found to be superior to the standard card of bulk ZnO with a hexagonal structure.

FESEM images of the ZnOTF that were presented in Fig. 2 show that the ZnOTF morphology appears to consist of interconnected spherical particles, but ZnOTF with 6 layers is more dense and homogenous than ZnOTF with 3 layers.

It is important to study the optical band structure and understand the nature of the transition also the band gap energy of the prepared ZnOTF. The absorbance spectra of the ZnOTFs displayed in Fig. 3a show high UV absorption characteristics at wavelengths less than 400 nm. The sharp absorption edge has been observed around 380 nm for the film with 3 layers was increased a little by increasing the layers of the ZnOTFs up to 6 layers.

For the prepared ZnOTFs, the standard transmittance and reflectance values were provided. The ZnOTFs’ absorption coefficient (α) and band gap (Eg) were found. The absorption coefficient can be computed using a simple approach from transmittance spectra by applying the following equation:

2 $$T = e^{akd}$$

where d is the film’s thickness and T is the optical transmittance. When the number of thin film layers was raised from three to six, as depicted in Fig. 3b, the absorption coefficient around the absorption edge (Eg) slightly increased at short wavelengths. This could be a result of the film becoming thicker.

Tauc plot shows the relation between (αhυ)^2^ and hυ and one could expect the Energy band gap from that plot as presented in Fug.3c.

It is noticed from the previous figure that as the film thickness increases, the optical band gap shows a tiny blue shift. This difference could be explained by the films’ quality changing as a result of more coatings giving rise to higher film thickness. The obtained results indicated that the ZnOTFs energy band gap, is 3.19 and 3.21 eV for ZnOTFs with 3 layers and 6 layers, respectively, with confirmed direct allowed electronic transition.

To confirm that the decrease in the absorption band comes from the effect of the ZnOTF in the presence of the UV light or it is only the action of the UV light and the prepared ZnO has no effect, a separate beaker filled with 25 ml of M.B. without any immersion of anything and irradiated with the UV lamp for 24 h and the used laser setup for 10 h as control sample it is noticed as it is clear in Fig. 4 that there is nearly no effect for the UV light without ZnO.

Figure 5 shows two main absorption bands. The highest band resonating at 664nm could be attributed to the *n* → π^∗^ transition that transition takes place by the free doublet on N of the C=N bond and the free doublet of S on the S=C bond. The shoulder at 612 nm could be attributed to the M.B. dimer formation. Also, the lowest bands at 292nm and 245 nm could be attributed to π → π ∗ transition that takes place in the substituted Benzene ring. The decrease of any of those bands represents the decay of the corresponding bond, i.e., degradation of the structure of some M.B. molecules. As decreasing the peak height, more degradation of the M.B. molecules takes place. It is noticed that the degradation was very slow. This could be attributed to the aggregation of the M.B. (as described in the introduction) molecules according to the high concentration of the prepared M.B. solution.

To avoid the alteration of the dimer formation, it is better to monitor the emission of the M.B. in this regime; it is better to use a laser source as a monochromatic source to excite the main M.B. band. A laser line with wavelength 635 nm has been utilized. The obtained fluorescence spectra have been presented in Fig. 6.

It is very important to study the stability of the prepared thin films and their cyclic usage several times over a long time to see if it is available to store for a long time or if they should be freshly prepared. For this purpose, the experiment was repeated for a single point with the same concentration after storing the samples at 4 °C for about a year. It is clear that the change is very little as it decreases about 2.5% and it could be considered the same as it comes in the range of error of the spectrophotometer.

It is clear from the photo-luminescence spectra that it confirms the extracted data from the absorption spectra and also confirms that the dimer formation did not alter the absorption data. Also, it confirms that the balance between adsorption and desorption has been reached before starting the Irradiation and photo-degradation process.

As an attempt to increase the degradation speed and reduce the degradation time UV line from Argon ion laser has been utilized instead of the UV lamp in a dark room. In this regime, a 305 nm laser line with power 5 mW has been expanded from 5 mm diameter to 60 mm diameter to cover the whole area of the ZnOTF layers. In this regime glass substrates coated by 6 layers of ZnO have been utilized in beakers filled with 25 ml M.B. for the same used concentration. 2ml of the solution was withdrawn after immersing the ZnOTF for 5 min, 10 min, 30 min, 60 min, 120 min, 180 min, and 300 min. It is noticed that the absorbance of both 180 min and 300 min did not change, as is clear in Fig. 6. Moreover, the absorbance at 180min and 300 min reached the absorbance value after 24h in the case of using a UV-lamb system; for that reason, we did not irradiate for more time. Figure 7 shows a little bit of fast degradation in the first 5 min and a monotonic increase till saturation after 180 min. Comparing the degradation rates of both UV lamb irradiated system and laser irradiated system one could easily notice the fast degradation in case of irradiating with the UV-laser. The increasing degradation rate could be explained as the laser irradiation breaking the M.B. dimers and the dissociation process takes place. The matter switches the M.B. from aggregated dimers to separate single molecules, and the M.B. molecules transform from inactive molecules to active molecules; this activation guides the ZnOTF with the help of UV energy do the degradation process.

In addition, it is observed that the laser irradiation affects more the benzene rings and increases the degradation of $\pi \to \pi^{*}$ bonds which, could be noticed by the decrease in the 299nm band as presented in Table 1.

**Table 1:** The peak height of the bands at 664 nm and 292 nm for the systems irradiated with UV-lamb and UV-laser.

Time (min)	Peak height (a.u.)					
	Three-layer UV lamb		6-layer UV lamb		6-layer UV laser	
	292 (nm)	664 (nm)	292 (nm)	664 (nm)	292 (nm)	664 (nm)
0	0.59	1.05	0.59	1.05	0.58	1.05
5	0.58	1.02	0.59	1.02	0.53	0.92
10	0.57	1.013	0.58	1.01	0.4	0.71
30	0.56	1.014	0.54	0.98	0.28	0.51
60	0.53	0.94	0.53	0.94	0.22	0.39
120	0.52	0.92	0.5	0.88	0.2	0.34
180	0.51	0.89	0.45	0.79	0.18	0.28
300	0.49	0.82	0.45	0.73	0.16	0.28
1440	0.41	0.51	0.26	0.28	–	–

Comparing the dimer band at 612 nm in both irradiation cases, one could easily notice that the laser irradiation affects the dimer band and decreases it in comparison with that irradiated with the UV lamb. Table 2 presents the decay in the dimer band moreover, the ratio between the monomer band at 664 nm and that of the dimer band at 612 nm.

**Table 2:** The peak height of the bands at 664 nm and 612 nm, and the ratio between both of them for the systems irradiated with UV-lamb and UV-laser.

Time (min)	UV lamb			UV laser		
	664 (nm)	612 (nm)	Ratio	664 (nm)	612 (nm)	Ratio
0	1.05	0.564	1.86	1.05	0.564	1.86
5	1.02	0.554	1.84	0.92	0.496	1.88
10	1.01	0.545	1.85	0.71	0.377	1.90
30	0.98	0.525	1.87	0.51	0.273	1.89
60	0.94	0.509	1.85	0.39	0.209	1.94
120	0.88	0.478	1.84	0.34	0.186	2.04
180	0.79	0.432	1.83	0.28	0.16	2.04
300	0.73	0.422	1.73	0.28	0.16	2.11

To understand the importance of this work it is better to make such a comparison with previous work. As it is clear in Table 2 the degradation reaches around 75% in only 180 min without any complicated system or any reactors as done by S. Mondal et. al.^41^ which degrade around 15% of the total M.B. concentration.

In photo-catalysis, two important factors should be calculated for every new photo-catalytic agent: the colour removal efficiency (η) and the apparent reaction constant, which controls the degradation kinetics reaction (k). Regarding the Color removal efficiency, it could be calculated using the following equation^37^

3 $$\eta = \frac{{C_{o} - C}}{{C_{o} }}$$

In the previous relation C_o_ and C are the zero time concentration and the last one for the M.B.

In addition, the degradation kinetics reaction could be calculated from initial and final concentrations, depending on the time taken to do the degradation (t). That kinetics are controlled by the apparent first-order kinetics, which could be known from the following exponential equation^37^:

4 $$C = C_{o} e^{kt}$$

To calculate the final concentration, four M.B. solutions with different concentrations have been prepared and measured, and a calibration curve has been plotted. Using the calibration equation, the concentration of every sample has been calculated and tabulated in Table 3; it is enough to calculate the mentioned factors using line 664 nm.

**Table 3:** The concentration of the withdrawn samples for the band 664nm.

Time (min)	Conc.(µM)		
	3 layers UV lamp	6 layers UV lamp	6 layers UV laser
0	14.81	14.81	14.81
5	14.46	14.46	13.31
10	14.38	14.35	10.88
30	14.39	14.0	8.57
60	13.54	13.54	7.19
120	13.31	12.85	6.61
180	12.96	11.81	5.92
300	12.15	11.11	5.92
1440	8.57	5.92	–

Applying Eq. 3, one could calculate the color removal efficiency (η), moreover, the apparent first-order kinetics (k) from Eq. 4; for the three systems as it is presented in Table 4.

**Table 4:** Colour removal efficiency (η), moreover the apparent first-order kinetics (k) for the three systems.

System	η (%)	k (min^− 1^)
3 Layers irradiated with UV lamb (after 24 h)	42	3.7*10^− 4^
6 Layers irradiated with UV lamb (after 24 h)	60	6.4*10^− 4^
6 Layers irradiated with UV laser (after 3 h)	60	5.1*10^− 3^

The used substrate area was constant for all experiments; it was 6.25 cm^2^. This means if the area gets doubled, one could get approximately more than 90% removal efficiency for the 6 layers within only 3 h if the sample was irradiated with a UV-laser. The benefit of this approach is that it did not involve the use of additional chemicals like H_2_O_2_^42^ or any other chemicals to achieve this level of removal. Molecular modelling could be used to understand the mechanisms of chemical reactions^50^, so to understand the presented photo-catalytic reaction mechanism, hydrated M.B. monomer, and Dimer in the presence/ absence of the ZnO have calculated as it is presented in Fig. 8. The optimized structure of the M.B. dimer shows the dimer formation according to hydrogen bonding between the monomer Nitrogen, sulfur, and zinc. Breaking this dimer depends on the energy of incident waves if it is higher than the band gap energy. The calculated band gap energy and total dipole moment of studied Zinc oxide (ZnO), M.B. hydrated with one water molecule (MB.H_2_O), M.B. hydrated with one water molecule interacted with ZnO (MB.H_2_O.ZnO), M.B. dimer hydrated with two water molecules (2MB.2H_2_O) and M.B. dimer hydrated with two water molecules interacted with ZnO (2MB.2H_2_O.ZnO) could be monitored from Table 5.

**Table 5:** The calculated total dipole moment (TDM) as Debye, HOMO/LUMO band gap energy (∆E) as eV for the investigated structures.

Structure	TDM (Debye)	∆E (ev)
ZnO	5.940	2.510
MB.H_2_O	6.372	1.368
MB.H_2_O.ZnO	9.910	0.695
2MB.2H_2_O	8.190	2.193
2MB.2H_2_O.ZnO	11.840	1.314

It is clear from Table 5 that the presence of ZnO decreases the Band gap energy of the M.B. dimer from 2.193 eV to 1.314 EV. To calculate the amount of laser power that hits the dimer molecules, the approximate dimer cross section should be calculated; here comes another time: the importance of the calculations.

According to the presented dimer structure in Fig. 7 (e), if we consider the dimer molecule as is surrounded by water molecules forming a hydrated dimer sphere. In this case, the hydrodynamic diameter of the M.B. dimer is nearly the distance between the H83 (the hydrogen atom of the water molecule to the left of the structure) and H77 (the hydrogen atom at the beginning of the hydrocarbon chain at the right of the structure) which equals approximately 1.46 nm. Hence, the projection of the hydrated dimer will be approximately 1.68 nm^2^.

In the experiment section, the power density has been calculated to be 0.25 mW/cm^2^; this means that every 1 cm^2^ exposed to 0.25 mW of UV laser, hence every 1 nm^2^ has been exposed to 2.5 × 10^− 15^ mW. According to the previous calculations, every hydrated M.B. dimer has been exposed to approximately 4.2 × 10^− 15^ mW or 26.2 eV, which is enough to break the dimer and initiate the photo-degradation reaction. According to Fig. 5, it is clear that the laser itself was not able to do the photo-degradation alone.

## Conclusion

ZnOTF had been prepared by spin-coating the sol–gel technique as 3 and 6 layers coated over the glass substrate surface. From the XRD, it was found that the prepared ZnOTF has a hexagonal structure. The surface morphology examination by FESEM proved the homogeneity of the prepared ZnOTF. The photo-catalysis experiment shows enhanced photo-degradation of M.B. for the 6-layer samples more than that coated with only 3 layers when exposed to a UV lamp in a dark room. Replacing the UV lambs with a 305 nm laser greatly enhances the degradation and overcomes the aggregated M.B. molecules due to the high concentration of the M.B. The kinetics and colour degradation efficiency proved that laser usage decreases the time of degradation. It is recommended to concentrate more on the UV laser irradiation by using different laser lines separately and in combination. Although the HOMO/LUMO band gap energy of ZnO (∆E = 2.510 eV), the irradiated ZnO interacted with MB dimer became more reactive as indicated by the HOMO/LUMO band gap energy (∆E = 1.314 eV) and total dipole moment (TDM = 11.840 Debye). The reactive behaviour of ZnO leads to the degradation of MB dimer into monomer.

## Data Availability

All data generated or analysed during this study are included in this published article.
